# Commutability of external quality assessment materials for point‐of‐care glucose testing using the Clinical and Laboratory Standards Institute and International Federation of Clinical Chemistry approaches

**DOI:** 10.1002/jcla.23327

**Published:** 2020-04-27

**Authors:** Yan Wang, Mario Plebani, Laura Sciacovelli, Shunli Zhang, Qingtao Wang, Rui Zhou

**Affiliations:** ^1^ Department of Laboratory Medicine Beijing Chao‐Yang Hospital, Capital Medical University Beijing China; ^2^ Department of Laboratory Medicine Padova University Hospital Padova Italy; ^3^ Beijing Center for Clinical Laboratories Beijing China

**Keywords:** blood glucose, commutability, external quality assessment, point‐of‐care testing, whole blood

## Abstract

**Objectives:**

The aim of this study was to assess the commutability of three external quality assessment (EQA) materials for point‐of‐care (POC) glucose testing using two approaches, to identify suitable EQA materials to evaluate and monitor the quality of POC testing.

**Methods:**

Commercial control materials (CCMs), pooled human serum samples (PHSs), and homemade human whole‐blood samples (HWBs) were measured along with 33 individual clinical samples using five POC instruments and a Hitachi 7600 analyzer. Data were analyzed by Deming regression analysis with a 95% prediction interval as described in Clinical and Laboratory Standards Institute (CLSI) EP30‐A, and by difference in bias analysis as described by the International Federation of Clinical Chemistry (IFCC) Working Group on Commutability.

**Results:**

Using the CLSI approach, HWBs, CCMs, and PHSs were commutable with five, one, and two instruments, respectively. With the IFCC approach, HWBs were commutable with two instruments, while CCMs and PHSs were largely inconclusive or non‐commutable on five instruments.

**Conclusions:**

HWBs were commutable on all instruments by the CLSI approach and may be a suitable EQA material for POC testing. Although some results differed between the IFCC and CLSI approaches, both indicated that HWBs were far superior to CCMs and PHSs in commutability.

## 1 INTRODUCTION

Point‐of‐care testing (POCT) is a popular means of providing laboratory testing at or near the site of patient care. It has become an important component of laboratory medicine by virtue of its portability and ease of operation by non‐laboratory personnel or patients themselves.^1^, ^2^, ^3^, ^4^, ^5^ Point‐of‐care (POC) glucose testing plays an important role in the treatment and management of diabetes mellitus, enabling strict glycemic control and creates opportunities to increase the efficiency of clinical services to improve patient outcomes.^6^, ^7^ Most analytical methods use one of three enzymatic reactions to quantify glucose: glucose oxidase (GOD), glucose dehydrogenase (GDH), or hexokinase/glucose‐6‐phosphate dehydrogenase (HK). In these systems, enzymatic activity produces an electrical current or color change proportional to the glucose concentration. Isotope dilution gas chromatography‐mass spectrometry (ID‐GC/MS) serves as a higher‐order procedure in reference laboratories, while the HK method is widely accepted for routine calibration and accuracy evaluation.^8^

Stringent accuracy assessment criteria for both self‐ and hospital‐based blood glucose monitoring have been proposed by many international organizations, including the International Standardization Organization (ISO) and the Clinical Laboratory and Standards Institute (CLSI).^9^, ^10^, ^11^, ^12^ However, in clinical application, the accuracy of POC glucose testing remains unsatisfactory. Several studies have described variability in measurements made by different POC glucose instruments or between these instruments and central laboratory analyzers,^13^, ^14^, ^15^, ^16^ mainly due to the lower specificity of the enzymes used (GOD and GDH), which make them susceptible to interference.^17^, ^18^

External quality assessment (EQA) is crucial to ensure the continuous high quality of medical laboratories. Commutability is required to be able to use EQA results to evaluate the performance of participating laboratories, as it enables measurement standardization. The International Vocabulary of Metrology defines the commutability of a reference material (RM) as close agreement between the measurements of a stated quantity of the material obtained by two different measurement procedures (MPs), as well as agreement between patient sample (PS) measurements. Miller et al have suggested an EQA scoring system with six categories, based on the ability of an EQA to evaluate participant and instrument performance.^19^ Category I is the most desirable, as programs in this category use commutable samples with target values established by a reference system, and can evaluate both individual laboratories and MPs for reproducibility, calibration traceability, and uniformity between laboratories and between MPs. Particularly for EQAs, the lack of commutability of applied samples is internationally recognized as one of the major hurdles in achieving a Category I POC glucose testing,^6^, ^19^ as it often impedes interpretation.^20^, ^21^

Because evaluating the commutability of EQA materials requires consistent sample typology (capillary samples) and stringent requirements that are difficult to apply, a pragmatic evaluation approach is required to ensure the correct interpretation of results provided in POC EQA reports. The aim of this study was to assess the commutability of three types of EQA materials by two different approaches, and to define suitable EQA materials to evaluate and monitor the quality of POC glucose testing.

## 2 MATERIALS AND METHODS

### 2.1 Study design

As EQA materials, we evaluated commercial control material (CCM), pooled human serum (PHS), and homemade human whole blood (HWB), all at three concentrations (denoted 1‐3), using five POC instruments and a laboratory‐based analyzer. The commutability of EQA materials was assessed by Deming regression analysis with a 95% prediction interval (PI), as described in CLSI EP30‐A^22^ and by bias difference analysis, as recommended by the International Federation of Clinical Chemistry (IFCC) Working Group (WG) on Commutability.^23^, ^24^

### 2.2 Experimental instruments

#### 2.2.1 Comparative instrument

A Hitachi 7600 Automatic Biochemical Analyzer (Hitachi Coro, Tokyo, Japan) was used as a comparative instrument, which uses the HK method (L‐Type Glu2, YZB/JAP 0915‐2003, Wako Pure Chemical Industries, Ltd.). This method is traceable to NIST standard material (SRM917) and is the generally accepted reference method for glucose measurement in central laboratories.^25^ The Hitachi 7600 analyzer is regularly involved in EQAs organized by the National Center for Clinical Laboratories (NCCL) in China, and its EQA results were satisfactory. Before experimentation, the analyzer was calibrated with a matched chemical calibrator (Batch No 999‐21401, Wako Pure Chemical Industries, Ltd.).

#### 2.2.2 POC glucose instruments

Five different mainstream‐brand POC glucose instruments were evaluated in this study (Table 1). Each POC instrument was operated and performed according to the specifications of its manufacturer. We performed one run with each instrument using one lot of strips and internal control materials, and these measurements were within the specified limits, indicating that all instruments were stable throughout the analysis period.

**Table 1:** Glucose POCT instruments tested and their manufacturers’ reported analytical performance parameters

Instrument	Manufacturer	Principle	Reportable range, mmol/dL	Blood sample	Hematocrit, %	Lot
ACCU‐CHEK Performa	Roche Diagnostics	GDH	0.6‐33.3	C, V, A, N	10.0‐65.0	474910
ACCU‐CHEK Active	Roche Diagnostics	GDH	0.6‐33.3	C, V, A, N	20.0‐70.0	23472431
StatStrip Xpress	Nova Biomedical	GOD	0.6‐33.3	C, V, A, N	No interference	0317248249
CONTOUR TS	Bayer Vital GmbH	GDH	0.6‐33.3	C, V, A, N	0.0‐70.0	DW6BM3E05B
HORIBA LP‐150C	HORIBA STEC, Co.	GOD	0.6‐55.5	C, V, A, N	20.0‐60.0	657021

Abbreviations: A, arterial; C, capillary; GDH, glucose dehydrogenase; GOD, glucose oxidase; N, neonate; V, venous.

### 2.3 Samples

#### 2.3.1 Individual PSs

The 33 venous blood samples (K_2_‐ethylenediaminetetraacetic acid (EDTA) anticoagulated) were obtained from residual clinical samples in the Laboratory Department of Beijing Chao‐Yang Hospital, the Third Clinical Medical College of Capital Medical University (Beijing, China), and included individuals with and without diabetes mellitus. Plasma glucose concentrations ranged from 3.19 to 21.94 mmol/L. Samples from patients with anemia, sepsis, and shock, and samples that were turbid, icteric, and hemolytic were excluded. Each PS was split into two aliquots and stored no longer than 2 hours at 2‐8°C prior to measurement. One aliquot was analyzed with the five POC instruments, as all five manufacturers state that their instruments are suitable for use with venous whole‐blood samples. The other aliquot was immediately centrifuged at 1600 *g* for 5 minutes to isolate the plasma for analysis on the Hitachi 7600. No significant interference from the EDTA was observed with any instrument.

#### 2.3.2 CCM

Low‐, medium‐, and high‐concentration CCMs (2.0‐4.0, 5.0‐12.0, and 13.0‐20.0 mmol/L, respectively) were prepared and provided by Guangzhou WONDFO Biotech Co., China. The aqueous CCMs were composed of water, glucose, and human hemoglobin, and were aliquoted (0.3 mL/tube) and stabilized at 2‐8°C for 2 weeks prior to experimentation. The homogeneity and stability of the materials were evaluated according to ISO 13528.^26^

#### 2.3.3 PHS

The PHSs were prepared by pooling serum samples collected from residual clinical serum samples in the Laboratory Department of Beijing Chao‐Yang Hospital. The inclusion and exclusion criteria for individual serum samples were the same as those for PSs. PHSs of low‐, medium‐, and high glucose concentration (<3.5, 4.0‐6.0, and 10.0 mmol/L, respectively) were collected into 50 mL test tubes. The serum pools were thoroughly mixed by inverting, aliquoted (0.3 mL/tube), and stored at 2‐8°C for 2 weeks. Exposure to freeze‐thaw cycles was limited to one cycle after serum collection and one cycle after pooling the sera. The homogeneity and stability of the materials were evaluated according to ISO 13528.^26^

#### 2.3.4 HWB (Patent No: 201811242371.5)

The HWBs were prepared by pooling ABO‐compatible EDTA whole‐blood samples collected from leftover clinical samples in the Laboratory Department of Beijing Chao‐Yang Hospital. The inclusion and exclusion criteria for these blood samples were the same as those for PSs. To prepare HWBs, whole‐blood samples were pooled (10 mL/tube) and allowed to undergo glycolysis overnight at 25°C to achieve a glucose concentration near zero. Next, the pooled samples were centrifuged at 1600 *g* for 5 minutes to separate the blood cells from the plasma. A 50% glucose solution was added to the separated plasma pools to produce final concentrations of 6.0, 16.0, or 28.0 mmol/L. The separated cells were fixed in a 4.0% formaldehyde and 4.0% glutaraldehyde solution for 24‐48 hours at 25°C, followed by three washes with 0.9% sodium chloride, filtering, and a final centrifugation at 1600 *g* for 5 minutes to pellet the fixed cells. Finally, the fixed cells and the plasma pools were recombined at 1:1 ratio to generate 3.0, 8.0, and 14.0 mmol/L HWBs. The samples were aliquoted (0.3 mL/tube) and stored at 2‐8°C for 2 weeks. The homogeneity and stability of the materials were evaluated according to ISO 13528.^26^

#### 2.3.5 Ethics statement

Because the study used anonymized leftover clinical samples, it did not require the consent of an ethical committee or review board.

### 2.4 Measurements

PSs and the three EQA materials were measured with five POC instruments and the Hitachi 7600 analyzer on the same day. All samples were adequately mixed at room temperature before analysis and measured in triplicate; for the EQA materials, three replicates were performed on each instrument. Samples were evaluated by the instruments in a set order, and the elapsed time between the first and last measurements was <30 minutes. All measurements were performed in a laboratory setting with controlled room temperature (23 ± 5°C) and humidity, according to the manufacturers’ specifications.

### 2.5 Data analysis

Microsoft Excel 2013 (Microsoft) was used to process the data, using formulas provided in the CLSI EP30‐A and IFCC WG on Commutability documents. Outlier values were excluded based on CLSI EP30‐A section 6.3.5: exclusion of data and handling of outliers in Part 2 of the IFCC document.^22^, ^24^ Of the 33 PSs, 30 were suitable for statistical analysis.

#### 2.5.1 Precision and comparability of different instruments

To evaluate the precision of each POC instrument, within‐run coefficients of variation (CVs) were calculated using triplicate measurements of PSs. Passing‐Bablok regression analysis was used to estimate the slopes and intercepts of each of the POC instruments vs the Hitachi 7600 analyzer, and the Spearman rank correlation coefficient was also calculated.

#### 2.5.2 Commutability assessment

Two different approaches were used for commutability evaluation. Difference plots were generated separately for comparisons between each POC instrument and the Hitachi 7600, and logarithm‐transformations were determined if scattering increased with concentration.

1. According to CLSI EP30‐A, the log_10_‐transformed results of PSs were analyzed by Deming regression analysis. A 95% PI around this regression line was calculated using the formulas described in CLSI EP30‐A Appendix C and was plotted along with the log_10_‐transformed results of the three EQA materials. When the result of each EQA material fell within the 95% PI it was regarded as commutable; otherwise, it was considered non‐commutable.^22^ As the materials in this study are used as EQAs, we have defined results touching the PI as commutable.

2. According to the recommendations of the IFCC WG on Commutability, a difference in bias approach was used. In this approach, the bias of each PS, *B*_ln (PSi)_, was calculated as the difference between the ln‐transformed mean results obtained with each POC instrument vs the Hitachi 7600 analyzer [ie, ln _(PSi, POC)_‐ln _(PSi,7600)_]. The mean bias of all PSs, ${\bar{B}}_{\ln(\;\text{PS)}}$ , *w*as used as an estimate of the bias for the PSs. The associated uncertainty, $u({\bar{B}}_{\ln(\text{PS})})$ , was calculated as the SD of the B_ln(Psi)_ values divided by the square root of the number of PSs (n = 30).

The bias of each EQA material, B_ln(Mj)_, was calculated as the difference between the ln‐transformed mean results obtained with each of the POC instruments vs the Hitachi 7600 analyzer [ie, ln_(Mj,POC)_‐ln_(Mj,7600)_]. To estimate the associated uncertainty of B_ln(M)_, the SDs between the replicate results of the EQA materials were pooled by calculating the mean variance for each POC instrument, ${\bar{\mathrm{SD}}}^{2}\left(\ln_{\left(M\right),\;\text{POC}}\right)$ , and for the Hitachi 7600, ${\bar{\mathrm{SD}}}^{2}\left(\ln_{\left(M\right),7600}\right)$ . ${\text{u(B}}_{\ln(\;\text{M)}})$ was calculated using the equation: $\sqrt{({\bar{\mathrm{SD}}}^{2}\left(\ln\left(M\right),\text{POC}\right)+{\bar{\mathrm{SD}}}^{2}\left(\ln\left(M\right),7600\right))/p}$ , in which *p* is the number of replicate measurements for each EQA material. The pooled SDs of the EQA materials assumed equal SDs, which were evaluated using a precision profile as described in Part 2 of the IFCC document.^24^

The difference in bias, D_Mj_, was estimated as $B_{\ln(Mj)}-{\bar{B}}_{\ln(PS)}$ . The associated expanded uncertainty U(D*_M_*) was calculated using the equation 1.9 $\times \sqrt{u^{2}\left(B_{\ln(M)}\right)+u^{2}\left({\bar{B}}_{\ln(\text{PS})}\right)}$ . The coverage factor 1.9 was used to obtain at least 90% coverage. To evaluate the commutability of an individual EQA material, the D*_Mj_* and U(D*_M_*) were compared with criterion C, which was set at 10.0% (1/2 of the desirable goal for the bias) based on ISO15197.^10^

In the comparability evaluation according to the IFCC WG approach, three outcomes were possible^27^, ^28^:

1. The uncertainty interval D_Mj_ ± U(D_M_) falls completely within 0 ± C → EQA M_j_ is commutable.
2. The uncertainty interval D_Mj_ ± U(D_M_) falls completely outside 0 ± C → EQA M_j_ is non‐commutable.
3. The uncertainty interval D_Mj_ ± U(D_M_) falls partially overlaps with 0 ± C → EQA M_j_ is inconclusive result.

Difference in bias (D_Mj_) and associated uncertainty (U(D_M_)) values can be found in the supplementary file.

## 3 RESULTS

### 3.1 Precision and comparability of different instruments

As shown in Table 2, the median within‐run CVs of the five POC instruments varied from 1.36% (the HORIBA LP‐150C) to 4.13 (the StatStrip Xpress). Passing‐Bablok slopes and intercepts and Spearman rank correlation coefficients for each POC‐Hitachi 7600 comparison are also shown in Table 2. The results from the five POC instruments showed good linear correlation, with Spearman coefficients ranging from 0.987 to 0.992. The slopes of the Passing‐Bablok regression lines varied from 0.891 to 1.166, and the intercepts varied from −0.385 to −0.065.

**Table 2:** Precision of each POC instrument and their correlations with the Hitachi 7600 analyzer using mean PS results

Instruments	Within‐run CV, % Median (Q1, Q3)	Intercept (95% CI)	Slope (95% CI)	Correlation coefficient
ACCU‐CHEK performa	2.91 (1.92, 4.05)	−0.066 (−0.339 to 0.254)	0.897 (0.841‐0.942)	.992
ACCU‐CHEK active	3.13 (1.34, 5.73)	−0.385 (−0.882 to 0.056)	1.166 (1.096‐1.255)	.987
StatStrip Xpress	4.13 (2.03, 6.23)	−0.227 (−0.462 to 0.074)	0.904 (0.858‐0.942)	.989
CONTOUR TS	3.82 (2.37, 5.08)	−0.087 (−0.349 to 0.162)	0.891 (0.850‐0.935)	.990
HORIBA LP‐150C	1.36 (0.49, 2.55)	−0.065 (−0.231 to 0.146)	1.002 (0.966‐1.027)	.991
Hitachi 7600	0.64 (0.44, 0.87)	N/A	N/A	N/A

Note

Regression parameters (slope and intercept) between each POC instrument and the Hitachi 7600 analyzer were calculated by Passing‐Bablok regression analysis.

Abbreviations: CI, confidence interval; CV, coefficient of variation; N/A, not applicable.

### 3.2 Commutability of the EQA materials according to the CLSI approach

**Figure 1:**
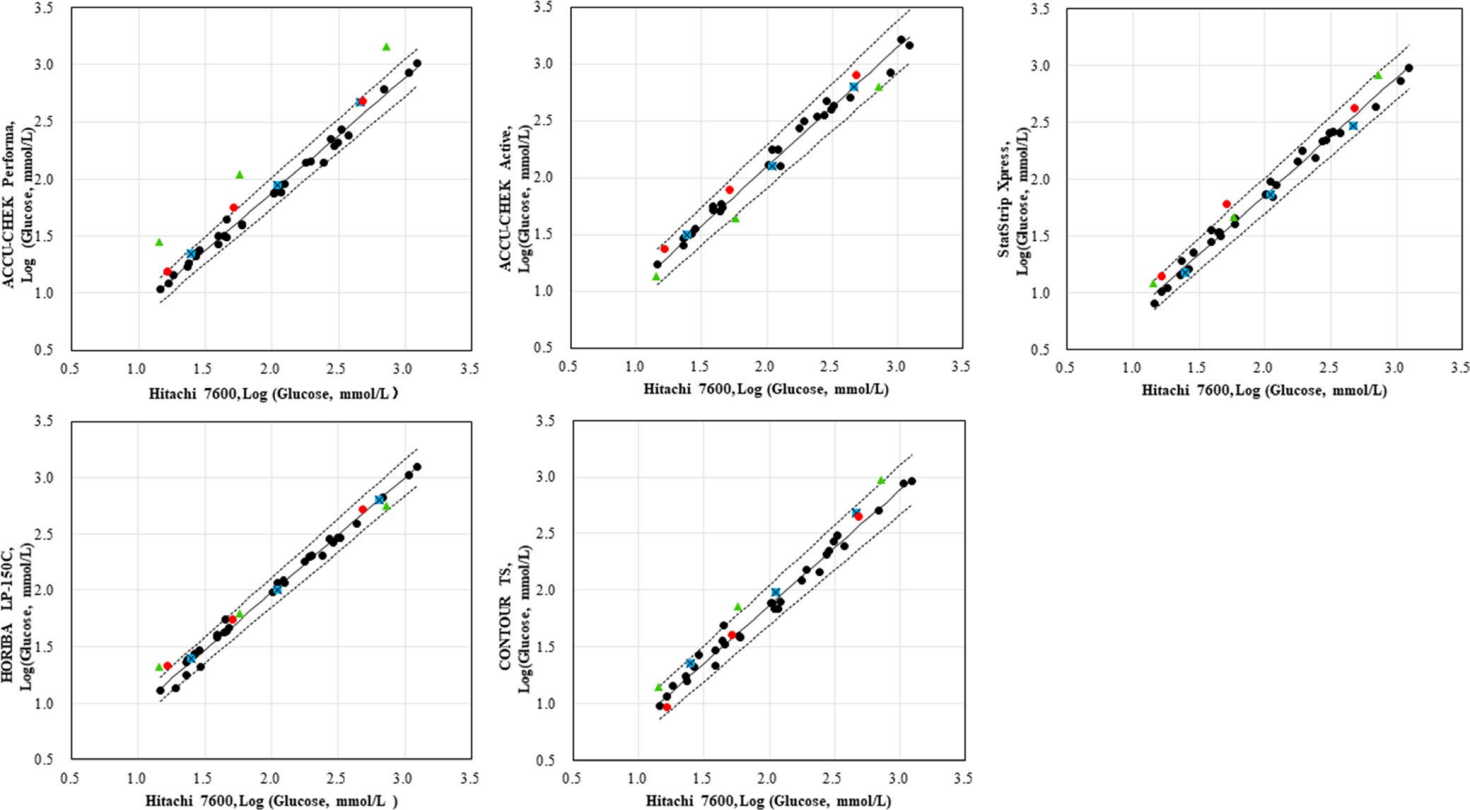
Commutability of the three EQA materials using the CLSI approach. Commutability assessment of the three external quality assessment (EQA) materials (commercial control materials (CCMs), pooled human serum samples (PHSs), and homemade human whole‐blood samples (HWBs) according to Clinical and Laboratory Standards Institute (CLSI) EP30‐A.^22^ The glucose levels of the EQA materials and patient samples (PSs) were measured with five point‐of‐care (POC) instruments and a Hitachi 7600 analyzer. The log‐transformed results measured by the Hitachi 7600 and the POC instruments are plotted on the x‐and y‐axes, respectively. Solid and dashed lines represent the regression lines and the limits of the 95% PIs of Deming regressions, respectively. The black circles represent the log‐transformed results of the PSs, and the blue squares, green triangles, and red circles represent the log‐transformed results of the HWBs, CCMs, and PHSs, respectively

Commutability assessments of the three EQA materials according to the CLSI approach are shown in Figure 1. CCM‐1, ‐2, and ‐3 were commutable on 3/5, 2/5, and 4/5 instruments, respectively. PHS‐1 ‐2, and ‐3 were commutable on 4/5, 3/5, and 5/5 instruments, respectively. HWBs at three concentrations were commutable on all five POC instruments, exhibiting the best performance among the three EQA materials by this approach.

### 3.3 Commutability of the EQA materials according to the IFCC approach

**Figure 2:**
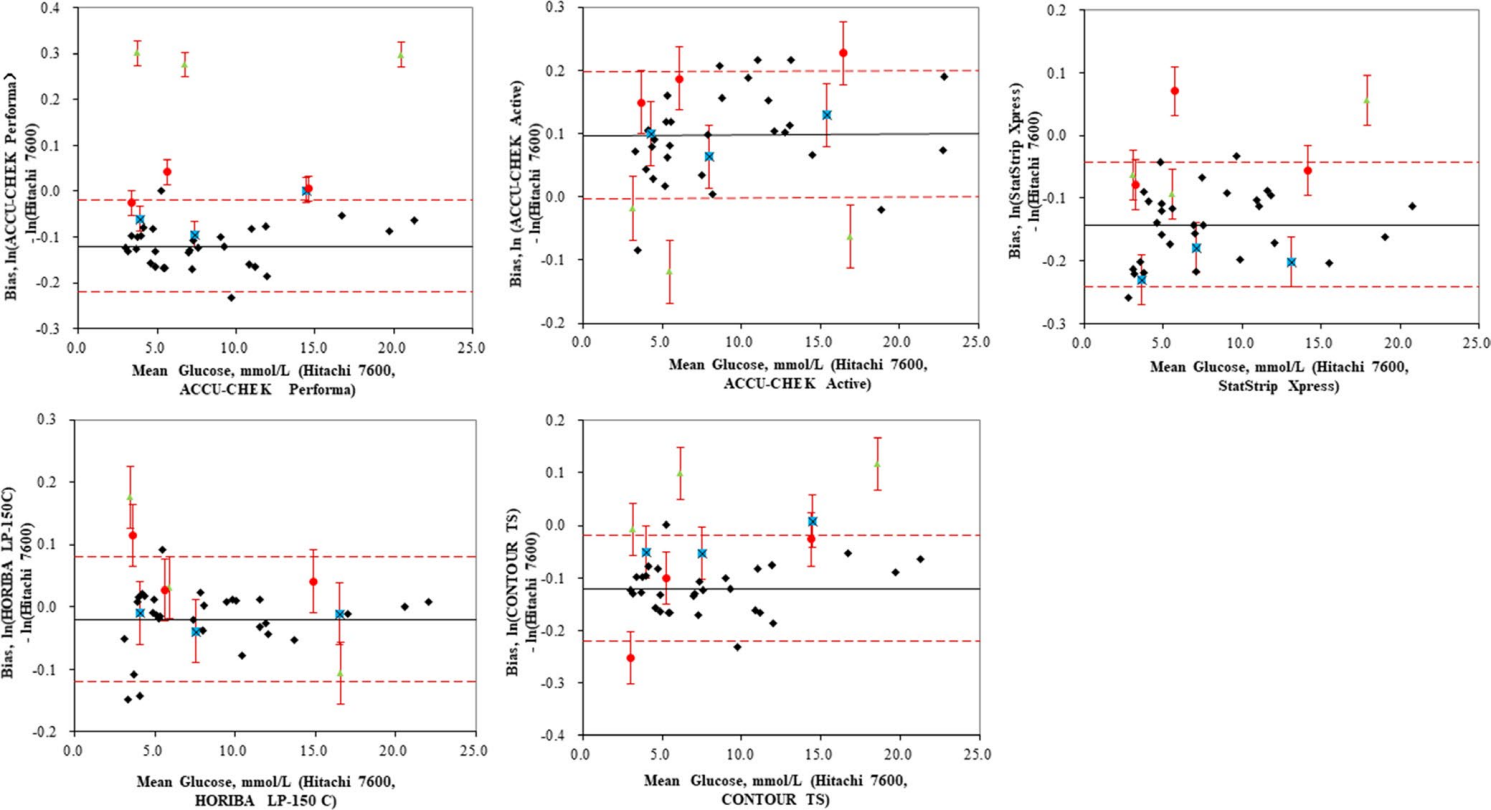
Commutability of the three EQA materials using the IFCC approach. Commutability assessment of the three external quality assessment (EQA) materials (commercial control materials (CCMs), pooled human serum samples (PHSs), and homemade human whole‐blood samples (HWBs) according to International Federation of Clinical Chemistry (IFCC) Working Group on Commutability.^23^, ^24^ The glucose levels of the EQA materials and patient samples (PSs) were measured with five point‐of‐care (POC) instruments and a Hitachi 7600 analyzer. The mean concentrations of each POC and the Hitachi 7600 are plotted on the x‐axis. The bias of the difference between the EQA materials and PSs is plotted on the y‐axis. The black solid lines represent the mean bias lines of the PSs, and the red dashed lines represent the commutability criteria. The black circles represent the bias of the PSs. The blue squares, green triangles, and red circles represent the mean bias between each POC and the Hitachi 7600 for the HWBs, CCMs, and PHSs, respectively. The red bars are the expanded uncertainty in the difference in bias between the EQA materials and the mean bias of the PSs

Commutability assessments of the three EQA materials according to the IFCC approach are shown in Figure 2. HWB‐1, ‐2, and ‐3 were commutable on 3/5, 4/5, and 3/5 instruments, respectively, while CCMs and PHSs were inconclusive or non‐commutable on all five POC instruments. All three HWB concentrations were commutable on the ACCU‐CHEK Performa and HORIBA LP‐150C.

### 3.4 Comparative commutability of the EQA materials using the two different approaches

Table 3 summarizes the individual results for each EQA material and each POC instrument according to the CLSI and IFCC approaches. Approximately 47% of the results were consistent between the two approaches, while 47% were inconsistent (commutable vs inconclusive or non‐commutable vs inconclusive). The CCM‐3 results were particularly inconsistent, as they were commutable on three POC instruments using the CLSI approach, but produced non‐commutable results using the IFCC approach.

**Table 3:** Commutability of HWBs, CCMs, and PHSs with five POC instruments compared to the Hitachi 7600 analyzer using two approaches

Material	Instrument									
	ACCU‐CHEK Performa		ACCU‐CHEK Active		StatStrip Xpress		HORIBA LP‐150C		CONTOUR TS	
	A	B	A	B	A	B	A	B	A	B
HWB‐1	C	C	C	C	C	I	C	C	C	I
HWB‐2	C	C	C	C	C	C	C	C	C	I
HWB‐3	C	I	C	C	C	C	C	C	C	I
CCM‐1	NC	NC	C	I	C	I	NC	NC	C	I
CCM‐2	NC	NC	NC	NC	C	C	C	I	NC	NC
CCM‐3	NC	NC	C	NC	C	NC	C	I	C	NC
PHS‐1	C	I	C	I	C	I	NC	I	C	I
PHS‐2	NC	NC	C	I	NC	NC	C	C	C	C
PHS‐3	C	I	C	I	C	I	C	I	C	I

Note

A: Deming regression analysis with 95% prediction interval, as described in the Clinical and Laboratory Standards Institute (CLSI) EP30‐A.^22^ B: Difference in bias analysis, as described in the recommendations of the International Federation of Clinical Chemistry (IFCC) Working Group.^23^, ^24^

Abbreviations: C, commutable; CCM, commercial control material; HWB, homemade human whole blood; I, inconclusive; NC, non‐commutable; PHS, pooled human serum.

## 4 DISCUSSION

The use of POCT in laboratory medicine is evolving at an increasing rate, with progressively more medical treatment decisions made based on it. Therefore, it is crucial to conduct EQAs to assess the accuracy and clinical reliability of POCT.^29^ If an EQA is category I, the consistency of results between different measuring systems can be assessed using a true value, which would improve the harmonization and standardization of POCT. However, a main issue for EQA organizers is the scarcity of commutable EQA materials that are compatible with different POC instruments.^21^ This study aimed to assess the commutability of three EQA materials using five POC glucose instruments and a central laboratory platform through two different approaches, to identify EQA materials that are as similar to native PSs as possible.

Before assessing the commutability of the three EQA materials, we evaluated the precision and comparability of the different instruments with PSs. In terms of the allowable imprecision error of POC glucose testing, Skeie et al^30^ stated that a within‐run CV <5.0% meets the clinical needs of 75.0% patients, with the exception of those with hypoglycemia. In this study, the HORIBA LP‐150C had the best precision, and all five POC instruments were acceptable, with within‐run CVs <5.0%. The results also displayed good linear correlation in each comparison.

Commutability assessments of the three EQA materials were first performed using the CLSI EP30‐A approach, which analyzes samples with the pair MPs and determines if the materials fall within the 95% PI. Bukve et al^31^ recently demonstrated that whole‐blood EQA material was commutable on three POC glucose instruments using this approach. Our study showed that HWBs were commutable on all five POC instruments at all three concentrations analyzed, while CCMs and PHSs were commutable on one and two instruments at all three levels, respectively. The CLSI approach is commonly used in RM commutability assessment^32^, ^33^; however, it has some limitations. First, the 95% PI is determined by how well correlated the analytical performances of the compared methods are, and more scatter in the relationship can easily make a material commutable. In other words, a RM can be commutable using a method with poor analytical performance but non‐commutable using a method with good analytical performance. Second, this approach depends on visual inspection of where the data points for each material are located in relation to the PSs and the limits of the PI. The approach provides no advice on how to interpret result points that are located on the limits of the PI.^23^ Therefore, the CLSI approach may not be ideal for assessing the commutability of EQA materials.

Difference in bias analysis to evaluate commutability was recently recommended by the IFCC WG to overcome the drawbacks of the CLSI approach. The IFCC approach determines whether the difference in bias between samples plus the uncertainty fulfills a fixed criterion to conclude whether a material is commutable, non‐commutable, or indeterminate. It quantifies the closeness of agreement and the associated uncertainty between RMs and clinical samples. The fixed commutability criterion is based on clinical application requirements and the intended use of a RM. Generally, for a material used as a trueness control in calibration traceability, the criterion should be strict in commutability validation, whereas for an EQA program, the criterion might be less stringent.^34^ As currently, most glucose POC instruments have decreased precision and lower accuracy in the hypoglycemic range than central laboratory analyzers,^13^, ^14^ the commutability criterion was set at 10.0%. Using this approach, HWBs were commutable with 2/5 POC instruments at all three concentrations, while the CCMs and PHSs were largely inconclusive or non‐commutable on all five POC instruments. These results indicate that HWBs have higher commutability than CCMs and PHSs.

Recent studies have reported different conclusions for the commutability assessment of EQA materials using these two approaches.^27^, ^28^ Consistently, our study revealed several inconsistencies between the two approaches (Table 3). By the CLSI approach, all three concentrations of HWBs were commutable on all five POC instruments, but five inconclusive results were produced using the IFCC approach. The CCMs and PHSs displayed some commutability by the CLSI approach, while the results of the IFCC approach were inconclusive. These discrepancies might be due to the uncertainty of difference being too large to fulfill the commutability criterion. Excessive uncertainty could be caused by unsuitable experimental design (inadequate replicates and/or clinical samples) or poor precision and/or poor selectivity (large sample‐specific differences).^24^ In addition, poor commutability characteristics can be caused by the nature of the analyte and its matrix, and the concentration can also affect the uncertainty.^35^ Based on this study, we suggested increasing the number of replicate measurements of the EQA materials to reduce the uncertainty.

A major limitation of the study was using the Hitachi 7600 analyzer as the comparative method. According to the IFCC’s latest recommendations on commutability assessment, the results of each routine method should be compared with those obtained using a higher‐order reference method. Although the HK method is still listed as a reference method for glucose measurement, ID‐GC/MS may provide a better reference point.

In conclusion, compared to CCMs and PHSs, HWBs had better commutability characteristics with mainstream POC glucose instruments by two different approaches, indicating that they are suitable EQA materials to evaluate and monitor the analytical quality of POC glucose testing. Furthermore, the results suggest that the IFCC approach for commutability evaluation should be used when selecting EQA materials for POCT.

## CONFLICT OF INTEREST

The authors declare no conflict of interest.

## Supporting information

Supplementary Material
