# Supplementary material for: Commutability of external quality assessment materials for point‐of‐care glucose testing using the Clinical and Laboratory Standards Institute and International Federation of Clinical Chemistry approaches
Source: J Clin Lab Anal. 2020 Apr 27;34(8):e23327. doi: 10.1002/jcla.23327 (PMC7439342; doi:10.1002/jcla.23327)
Supplement: Supplementary file 1 [file JCLA-34-e23327-s001.docx]

Commutability of EQA materials															
Instrument															
x:	HITACHI 7600														
y:	ACCU-CHEK Performa														
Variable:	ln(concentration)														
Commutability criterion															
C =	0.1														C= Commutable
M:	EAQ material														
PS:	patient sample														
B:bias												Cov. factor=	1.9		NC= Non-commutable
EQA materials						PS				Commutability					I= Inconclusive
	Conc both	B(M)	Pooled	Pooled			B(PS)	sB	u(BPS)				Uncertainty limits for DM		
ID	(x+y)/2	ln(y)-ln(x	sPos-mean(x)	sPos-mean(y)	u(BM)	n				DM	u(DM)	U(DM)	Lower limit	Higher limit	Commutable?
HWB-1	3.94	-0.060	0.014	0.014	0.011	30	-0.120	0.047	0.009	0.060	0.014	0.027	0.033	0.087	C
HWB-2	7.36	-0.094	0.014	0.014	0.011	30	-0.120	0.047	0.009	0.026	0.014	0.027	-0.001	0.053	C
HWB-3	14.43	0.002	0.014	0.014	0.011	30	-0.120	0.047	0.009	0.122	0.014	0.027	0.095	0.149	I
CCM-1	3.72	0.301	0.014	0.014	0.011	30	-0.120	0.047	0.009	0.421	0.014	0.027	0.394	0.448	NC
CCM-2	6.74	0.276	0.014	0.014	0.011	30	-0.120	0.047	0.009	0.396	0.014	0.027	0.369	0.423	NC
CCM-3	20.46	0.297	0.014	0.014	0.011	30	-0.120	0.047	0.009	0.417	0.014	0.027	0.390	0.444	NC
PHS-1	3.34	-0.026	0.014	0.014	0.011	30	-0.120	0.047	0.009	0.094	0.014	0.027	0.067	0.121	I
PHS-2	5.65	0.042	0.014	0.014	0.011	30	-0.120	0.047	0.009	0.162	0.014	0.027	0.135	0.189	NC
PHS-3	14.63	0.006	0.014	0.014	0.011	30	-0.120	0.047	0.009	0.126	0.014	0.027	0.099	0.153	I

Instrument															
x:	HITACHI 7600														
y:	ACCU-CHEK Active														
Variable:	ln(concentration)														
Commutability criterion															
C =	0.1														C= Commutable
M:	EAQ material														
PS:	patient sample														
B:	bias											Cov. factor=	1.9		NC= Non-commutable
EQA materials						PS				Commutability					I= Inconclusive
	Conc both	B(M)	Pooled	Pooled			B(PS)	sB	u(BPS)				Uncertainty limits for DM		
ID	(x+y)/2	ln(y)-ln(x	sPos-mean(x)	sPos-mean(y)	u(BM)	n				DM	u(DM)	U(DM)	Lower limit	Higher limit	Commutable?
HWB-1	4.24	0.1	0.013848385	0.03746109	0.02	30	0.09653	0.0711	0.012985	0.0034667	0.0264634	0.050280537	-0.04681382	0.053747252	C
HWB-2	7.961666667	0.06399	0.013848385	0.03746109	0.02	30	0.09653	0.0711	0.012985	-0.0325467	0.0264634	0.050280537	-0.08282721	0.017733861	C
HWB-3	15.41666667	0.1291	0.013848385	0.03746109	0.02	30	0.09653	0.0711	0.012985	0.032566	0.0264634	0.050280537	-0.01771449	0.082846586	C
CCM-1	3.133333333	-0.0186	0.013848385	0.03746109	0.02	30	0.09653	0.0711	0.012985	-0.1151312	0.0264634	0.050280537	-0.16541171	-0.064850637	I
CCM-2	5.491666667	-0.1183	0.013848385	0.03746109	0.02	30	0.09653	0.0711	0.012985	-0.2148521	0.0264634	0.050280537	-0.26513265	-0.164571579	NC
CCM-3	16.90833333	-0.0628	0.013848385	0.03746109	0.02	30	0.09653	0.0711	0.012985	-0.1592893	0.0264634	0.050280537	-0.20956982	-0.109008747	NC
PHS-1	3.658333333	0.1492	0.013848385	0.03746109	0.02	30	0.09653	0.0711	0.012985	0.0526633	0.0264634	0.050280537	0.002382774	0.102943848	I
PHS-2	6.1	0.18702	0.013848385	0.03746109	0.02	30	0.09653	0.0711	0.012985	0.0904874	0.0264634	0.050280537	0.040206814	0.140767888	I
PHS-3	16.44333333	0.2269	0.013848385	0.03746109	0.02	30	0.09653	0.0711	0.012985	0.1303678	0.0264634	0.050280537	0.080087237	0.180648311	I

Instrument															
x:	HITACHI 7600														
y:	StatStrip Xpress														
Variable:	ln(concentration)														
Commutability criterion															
C =	0.1														C= Commutable
M:	EAQ material														
PS:	patient sample														
B:	bias											Cov. factor=	1.9		NC= Non-commutable
EQA materials						PS				Commutability					I= Inconclusive
	Conc both	B(M)	Pooled	Pooled			B(PS)	sB	u(BPS)				Uncertainty limits for DM		
ID	(x+y)/2	ln(y)-ln(x	sPos-mean(x)	sPos-mean(y)	u(BM)	n				DM	u(DM)	U(DM)	Lower limit	Higher limit	Commutable?
HWB-1	3.635	-0.23	0.014243907	0.02792695	0.02	30	-0.1419	0.0568	0.0103728	-0.088077	0.0208613	0.03963656	-0.12771358	-0.048440457	I
HWB-2	7.078333333	-0.1787	0.014243907	0.02792695	0.02	30	-0.1419	0.0568	0.0103728	-0.0367713	0.0208613	0.03963656	-0.07640782	0.002865301	C
HWB-3	13.1	-0.2021	0.014243907	0.02792695	0.02	30	-0.1419	0.0568	0.0103728	-0.0601463	0.0208613	0.03963656	-0.09978288	-0.020509762	C
CCM-1	3.066666667	-0.0635	0.014243907	0.02792695	0.02	30	-0.1419	0.0568	0.0103728	0.0784676	0.0208613	0.03963656	0.03883103	0.118104149	I
CCM-2	5.558333333	-0.0929	0.014243907	0.02792695	0.02	30	-0.1419	0.0568	0.0103728	0.0490064	0.0208613	0.03963656	0.009369797	0.088642916	C
CCM-3	17.94166667	0.05589	0.014243907	0.02792695	0.02	30	-0.1419	0.0568	0.0103728	0.1978093	0.0208613	0.03963656	0.158172754	0.237445873	NC
PHS-1	3.258333333	-0.078	0.014243907	0.02792695	0.02	30	-0.1419	0.0568	0.0103728	0.063908	0.0208613	0.03963656	0.024271435	0.103544554	I
PHS-2	5.733333333	0.07017	0.014243907	0.02792695	0.02	30	-0.1419	0.0568	0.0103728	0.2120945	0.0208613	0.03963656	0.172457896	0.251731015	NC
PHS-3	14.19333333	-0.0557	0.014243907	0.02792695	0.02	30	-0.1419	0.0568	0.0103728	0.0862213	0.0208613	0.03963656	0.046584704	0.125857823	I

Instrument															
x:	HITACHI 7600														
y:	HORIBA LP-150C														
Variable:	ln(concentration)														
Commutability criterion															
C =	0.1														C= Commutable
M:	EAQ material														
PS:	patient sample														
B:	bias											Cov. factor=	1.9		NC= Non-commutable
EQA materials						PS				Commutability					I= Inconclusive
	Conc both	B(M)	Pooled	Pooled			B(PS)	sB	u(BPS)				Uncertainty limits for DM		
ID	(x+y)/2	ln(y)-ln(x	sPos-mean(x)	sPos-mean(y)	u(BM)	n				DM	u(DM)	U(DM)	Lower limit	Higher limit	Commutable?
HWB-1	4.035	-0.01	0.014243907	0.04157116	0.03	30	-0.0187	0.0498	0.0090872	0.0087045	0.0269492	0.051203518	-0.04249907	0.059907971	C
HWB-2	7.561666667	-0.0388	0.014243907	0.04157116	0.03	30	-0.0187	0.0498	0.0090872	-0.0200591	0.0269492	0.051203518	-0.07126264	0.031144396	C
HWB-3	16.515	-0.0107	0.014243907	0.04157116	0.03	30	-0.0187	0.0498	0.0090872	0.0079875	0.0269492	0.051203518	-0.04321602	0.059191017	C
CCM-1	3.466666667	0.17519	0.014243907	0.04157116	0.03	30	-0.0187	0.0498	0.0090872	0.1938915	0.0269492	0.051203518	0.142687943	0.24509498	NC
CCM-2	5.908333333	0.03116	0.014243907	0.04157116	0.03	30	-0.0187	0.0498	0.0090872	0.0498666	0.0269492	0.051203518	-0.00133696	0.101070074	I
CCM-3	16.55833333	-0.1064	0.014243907	0.04157116	0.03	30	-0.0187	0.0498	0.0090872	-0.0876484	0.0269492	0.051203518	-0.13885188	-0.03644484	I
PHS-1	3.591666667	0.11477	0.014243907	0.04157116	0.03	30	-0.0187	0.0498	0.0090872	0.1334746	0.0269492	0.051203518	0.082271081	0.184678118	I
PHS-2	5.616666667	0.02732	0.014243907	0.04157116	0.03	30	-0.0187	0.0498	0.0090872	0.0460294	0.0269492	0.051203518	-0.00517411	0.097232923	C
PHS-3	14.89333333	0.04125	0.014243907	0.04157116	0.03	30	-0.0187	0.0498	0.0090872	0.0599564	0.0269492	0.051203518	0.008752887	0.111159924	I

Instrument															
x:	HITACHI 7600														
y:	C0NTOUR TS														
Variable:	ln(concentration)														
Commutability criterion															
C =	0.1														C= Commutable
M:	EAQ material														
PS:	patient sample														
B:	bias											Cov. factor=	1.9		NC= Non-commutable
EQA materials						PS				Commutability					I= Inconclusive
	Conc both	B(M)	Pooled	Pooled			B(PS)	sB	u(BPS)				Uncertainty limits for DM		
ID	(x+y)/2	ln(y)-ln(x	sPos-mean(x)	sPos-mean(y)	u(BM)	n				DM	u(DM)	U(DM)	Lower limit	Higher limit	Commutable?
HWB-1	3.951666667	-0.05	0.014243907	0.04484046	0.03	30	-0.1204	0.0467	0.0085183	0.0704029	0.0284678	0.054088745	0.016314143	0.124491632	I
HWB-2	7.511666667	-0.0529	0.014243907	0.04484046	0.03	30	-0.1204	0.0467	0.0085183	0.0674734	0.0284678	0.054088745	0.013384644	0.121562134	I
HWB-3	14.48333333	0.00814	0.014243907	0.04484046	0.03	30	-0.1204	0.0467	0.0085183	0.1285466	0.0284678	0.054088745	0.074457835	0.182635325	I
CCM-1	3.15	-0.008	0.014243907	0.04484046	0.03	30	-0.1204	0.0467	0.0085183	0.1123879	0.0284678	0.054088745	0.058299154	0.166476643	I
CCM-2	6.125	0.0983	0.014243907	0.04484046	0.03	30	-0.1204	0.0467	0.0085183	0.2186993	0.0284678	0.054088745	0.164610564	0.272788053	NC
CCM-3	18.54166667	0.11773	0.014243907	0.04484046	0.03	30	-0.1204	0.0467	0.0085183	0.2381376	0.0284678	0.054088745	0.184048842	0.292226331	NC
PHS-1	3.008333333	-0.2519	0.014243907	0.04484046	0.03	30	-0.1204	0.0467	0.0085183	-0.1315056	0.0284678	0.054088745	-0.1855943	-0.077416813	I
PHS-2	5.266666667	-0.1007	0.014243907	0.04484046	0.03	30	-0.1204	0.0467	0.0085183	0.0197074	0.0284678	0.054088745	-0.0343813	0.073796185	C
PHS-3	14.39333333	-0.0268	0.014243907	0.04484046	0.03	30	-0.1204	0.0467	0.0085183	0.0935955	0.0284678	0.054088745	0.039506743	0.147684233	I
